# Older Couples’ Advance Care Planning Engagement Patterns and Associations with Individual and Spousal Factors

**DOI:** 10.1093/geroni/igab046.2860

**Published:** 2021-12-17

**Authors:** Hyo Jung Lee, Bon Kim

**Affiliations:** 1 Nanyang Technological University, Singapore, Singapore; 2 Jeju National University, Jeju-si, Cheju-do, Republic of Korea

## Abstract

This study examines older couples’ dyadic patterns of informal and formal advance care planning (ACP) and determines the associations of these patterns with their own and spousal characteristics. Using data from the 2014 and 2016 Health and Retirement Study, we performed a) latent class analysis to identify distinctive ACP engagement patterns and b) multinomial regression models to describe related characteristics of older couples (N = 1,545 couples). We identified four dyadic patterns of ACP engagement: a) high ACP engaging couple (45%); b) high engaging husband – low engaging wife (13%); c) high engaging wife – low engaging husband (11%); and d) low engaging couple (31%). Engagement in informal and formal ACP was associated with both individual and spousal factors: Older couples with advanced age or higher levels of education and wealth were more likely to engage in both informal and formal ACP, whereas only wife’s high level of constrain or husband’s greater number of depressive symptoms was associated with discordant ACP engagements. Couple-based approach to promote ACP merits older couples with limited resources or poorer psychological health in both or either spouse.

